# An Orthogonal Test Study on the Preparation of Self-Compacting Underwater Non-Dispersible Concrete

**DOI:** 10.3390/ma16196599

**Published:** 2023-10-08

**Authors:** Haibin Geng, Huijuan Wang, Xiaoke Li, Lin Wang, Hao Zhong, Changyong Li

**Affiliations:** 1 Collaborative Innovation Center for Efficient Utilization of Water Resources, International Joint Research Lab for Eco-Building Materials and Engineering of Henan, North China University of Water Resources and Electric Power, Zhengzhou 450045, China; hbgeng@stu.ncwu.edu.cn (H.G.); wanglin20001002@stu.ncwu.edu.cn (L.W.); zhonghao20001222@stu.ncwu.edu.cn (H.Z.); 2 Low-Carbon Eco-Building Materials Technology Innovation Center of Xuchang City, Civil Engineering and Architecture School, Zhongyuan Institute of Science and Technology, Zhengzhou 450042, China; wanghuijuan1@zykjxy.wecom.work; 3 School of Architecture, North China University of Water Resources and Electric Power, Zhengzhou 450045, China

**Keywords:** underwater non-dispersible concrete, orthogonal test, workability, washout resistance, compressive strength

## Abstract

To ensure a limited washout loss rate and the self-compaction of underwater concrete, the mix proportion design of underwater non-dispersible concrete is a key technology that has not been completely mastered. In view of this aspect, an orthogonal test study was carried out in this paper on the workability, washout resistance, and compressive strength of underwater non-dispersible concrete. Six factors with five levels were considered, which included the water/binder ratio, the sand ratio, the maximum particle size of the coarse aggregate, the content of the dispersion resistance agent, the content of superplasticizer, and the dosage of fly ash. Using a range and variance analysis, the sensitivity and significance of these factors were analyzed on the slump and slump-flow, the flow time, the washout loss rate, the pH value, and the compressive strength at the curing ages of 7 days and 28 days. The results indicated that the water/binder ratio and the content of the dispersion resistance agent were strong in terms of their sensitivity and significance on the workability and washout resistance, and the water/binder ratio and the dosage of fly ash were strong in terms of their sensitivity and significance on the compressive strength. With the joint fitness of the test results, formulas for predicting the slump-flow, washout loss rate, and compressive strength of underwater non-dispersible concrete were proposed considering the main impact factors.

## 1. Introduction

With the development of oceanic exploration and water infrastructure construction, Underwater Non-dispersible Concrete (UNC) has become a special high-performance concrete [1,2,3]. It avoids the waste of resources with traditional cofferdam construction methods, and greatly improves economic efficiency. Furthermore, it is environmentally friendly, without pollution to the surrounding environment during its casting in water. Therefore, UNC has been used in various projects such as underwater tunnel grouting [4,5], artificial island construction [5], channel lining board repair [6], embankment reinforcement [7], underwater immersion piles [5,8], and cofferdam bottom sealing [9]. Due to its unique construction conditions, UNC requires a strong ability to resist washout and be self-compacted without vibration. In view of the dispersion resistance of fresh UNC, a dispersion resistance agent is always added to conventional concrete. Dispersion resistance agents include fiber ethers such as hydroxypropyl methylcellulose [10] and hydroxyethyl cellulose [11], acrylic acid polymers such as polyacrylamide [11,12,13], a polysaccharide flocculating agent such as Welan gum [14,15,16] and UWB-II [11,14], and other admixtures such as silica fume [12,13] and geopolymers [17]. However, the addition of a dispersion resistance agent often leads to a significant reduction in the workability of the fresh concrete, which is not conducive to underwater construction. Therefore, a challenge is always faced in achieving a balance between the washout resistance and good workability of UNC.

Currently, many studies have been conducted on the impacts of different components on the workability and washout resistance of UNC. Wang et al. [11] investigated the effect of aggregate gradation, the content of the water reducer, the water/binder ratio, and the content of the UWB-II dispersion resistance agent on the workability of UNC, and the results showed that an increase in the content of the dispersion resistance agent reduced the workability of fresh concrete, while increases in the aggregate particle size, the content of the water reducer, and the water/binder ratio increased the workability of fresh concrete. Wen et al. [16] reported that increasing the content of the dispersion resistance agent helps to improve the washout resistance of fresh concrete. Zhang et al. [18] investigated the slump and the suspension content of UNC affected by the water/binder ratio, the cellulose dispersion resistance agent, and the polycarboxylate water reducer, and the results showed that the factors affecting the slump of the UNC were in the order of the water/binder ratio, the content of the water reducer, and the content of the dispersion resistance agent; the factors affecting the washout resistance were in the order of the content of the dispersion resistance agent, the water/binder ratio, and the content of the water reducer. Zhang and Dong [12] investigated the effect of fly ash and silica fume on the workability and washout resistance of UNC using polyacrylamide as a dispersion resistance agent, and the results indicated that the washout resistance of concrete was sharply reduced when the content of fly ash exceeded 30% of the binders, while it was significantly improved by adding silica fume within 20% of the binders. Dong and Zhang [13] investigated the effect of the water/binder ratio, sand ratio, blended fly ash, and silica fume on the workability and washout resistance of UNDC using polyacrylamide as a dispersion resistance agent, and the results showed that an increase in the sand ratio and the addition of mineral admixtures reduced the flowability of the concrete, while increasing the water/binder ratio increased the flowability but reduced the washout resistance. Zhang et al. [19] studied the effect of the water/binder ratio, sand ratio, and content of the dispersion resistance agent on the workability and washout resistance of UNC using UWB-II as a dispersion resistance agent, and the results indicated that increasing the water/binder ratio improved the workability of the concrete but reduced its washout resistance. The workability was reduced but the washout resistance was improved using the dispersion resistance agent, and the best workability was observed at a sand ratio of 42%. Nasr et al. [20] studied the effect of the water/binder ratio, sand ratio, and water reducer on the workability, and the results showed that the workability of the concrete was improved but the washout resistance was decreased with an increase in the water/binder ratio and the content of the water reducer, with the optimal workability being observed at a sand ratio of 50%. Kumar et al. [21] investigated the effect of the blended use of fly ash and silica fume on the workability and washout resistance of concrete, showing that an increase in the dosage of silica fume reduced the flowability but improved the washout resistance of concrete, while fly ash exhibited the opposite trend. However, due to many factors’ influences on the performance of UNC, the method for obtaining the optimal mix proportion of UNC still needs to be investigated.

The research team of the authors of this paper investigated the influences of different compositions of recycled aggregates [22], high-volume fly ash and fine-grinding fly ash [23,24], and blended mineral admixture [25] on the workability, durability, and mechanical properties of concrete and its durability exposed in a severe environment [26]. This provides a foundation for investigating the preparation of self-compacting UNC. With reference to the above studies on UNC, the orthogonal test design method was used to plan a test for twenty-five groups of UNC with six factors at five levels. The six factors included the water/binder ratio, the sand ratio, the particle size of coarse aggregates, the content of the dispersion resistance agent, the content of the water reducer, and the dosage of fly ash. The indices measured included the slump and slump-flow, the flow time, the washout loss rate, the pH value, and the compressive strength of the UNC. The sensitivity and significance of each factor on the performance indicators of the UNC were analyzed using a range and variance analysis. The main factors influencing the properties of the UNC were determined to be used for building formulas for predicting the flowability, the washout loss rate, and the compressive strength of the UNC.

## 2. Materials and Methods

### 2.1. Raw Materials

Common Portland cement of a 42.5 strength grade with a density of 3050 kg/m^3^ and a specific surface area of 356 m^2^/kg was used. Class-II fly ash was applied for a mineral admixture with a density of 2250 kg/m^3^ and a specific surface area of 406 m^2^/kg. The chemical compositions of the cement and fly ash are summarized in Table 1. The properties of the cement and fly ash met the requirements of China codes GB 175 [27] and GB/T 50146 [28].

The coarse aggregate was continuous grading crushed limestone with an apparent density of 2720 kg/m^3^ and maximum particle sizes of 10 mm, 16 mm, 20 mm, 25 mm, and 31.5 mm, respectively. The fine aggregate was natural river sand with an apparent density of 2650 kg/m^3^ and a fineness modulus of 2.65. The properties of the aggregates were tested according to the methods specified in China code JGJ 52 [29].

The dispersion resistance agent was white powder UWB-II flocculating agent [11,16,19], which was produced by CNPC Engineering Technology Research Institute, Beijing, China. A fluid polycarboxylate superplasticizer was also used with a density of 1.05 kg/L and a water reduction of 25%.

The mix water was the city tap water of Zhengzhou, China.

### 2.2. Orthogonal Test Design

Based on the analyses of the main factors in previous studies, six factors, including the water/binder ratio (*w*/*b*), the sand ratio (*β*_s_), the maximum particle size of the coarse aggregates (*d_c_*_a_), the percent of the dispersion resistance agent in the mass of the binders (*P*_DRA_), the percent of superplasticizer in the mass of the binders (*P*_SP_), and the percent of the fly ash in the mass of the binders (*P*_FA_), are presented in Table 2.

An orthogonal test was designed on 5 levels, and a total of 25 mixed experiments were designed using the standard design sheet L25 (5^6^) presented in Table 3.

### 2.3. Test Methods

With the selected parameters, the mix proportion of the UNC was designed using the absolute volume method, which was applied for the self-compacting concrete [30,31].

According to the specifications of China codes GB/T 50080 [32] and JGJ/T 283 [33], tests for slump flowability were conducted to measure the slump (*S*), the slump-flow (*SF*), and the flow time (*T*_sf_) of the fresh UNC. The slump test measured the height at which the concrete mixture collapsed under self-weight. The *SF* refers to the flowing diameter of the concrete mixture after slumping. The *T*_sf_ is the time for the concrete mixture to reach a certain diameter after slumping, which is marked as *T*_500_ and *T*_400_, corresponding to diameters of 500 mm and 400 mm, respectively.

**Figure 1:**
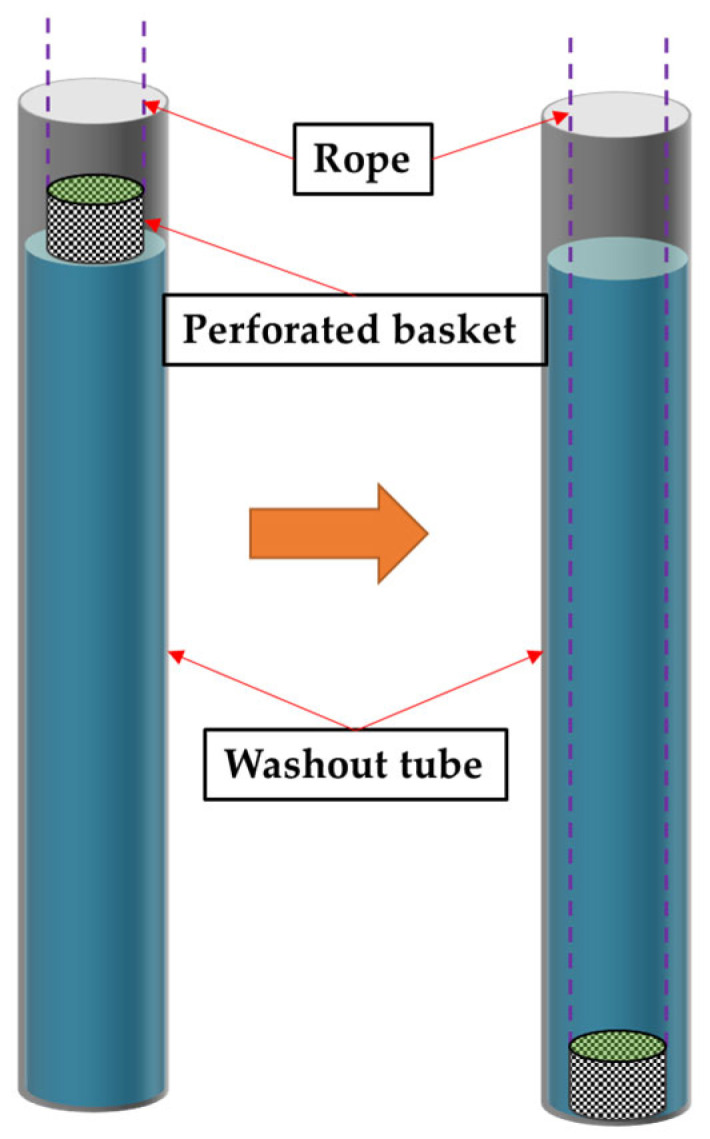
Devise for washout loss test of UNC.

Testing of the washout loss (*M*_loss_) and pH value of the UNC was conducted in accordance with USA CRD C61 [34] and China code DL/T 5117 [35]. The anti-dispersion testing device is shown in Figure 1, in a tube with water of a 1.7 m height. A fresh concrete sample in a perforated basket freely fell to the bottom of the tube and was left to stand for 15 s, then the sample was retrieved at a constant speed of 0.5 m/s, and the mass loss due to the washout was measured. The washout losses reported are the cumulative of three tests of the sample in the water. Ten equal parts divided from 500 g of fresh UNC were subsequently placed into an 800 mL beaker filled with distilled water and stood for 3 min, and the water sample from the top layer was taken for the testing of the pH value. The compressive strength (*f*_cu_) of the UNC was measured at the curing ages of 7 days and 28 days using cubic specimens with dimensions of 100 mm, according to the specification of China code JGJ/T283 [33].

## 3. Results and Discussion

### 3.1. Results of the Orthogonal Test

The rest results of the workability (*S*, *SF*, and *T*_sf_), the anti-dispersion performance (*M*_loss_ and pH), and the compressive strength (*f*_cu,7_ and *f*_cu,28_) of the UNC are summarized in Table 4.

**Figure 2:**
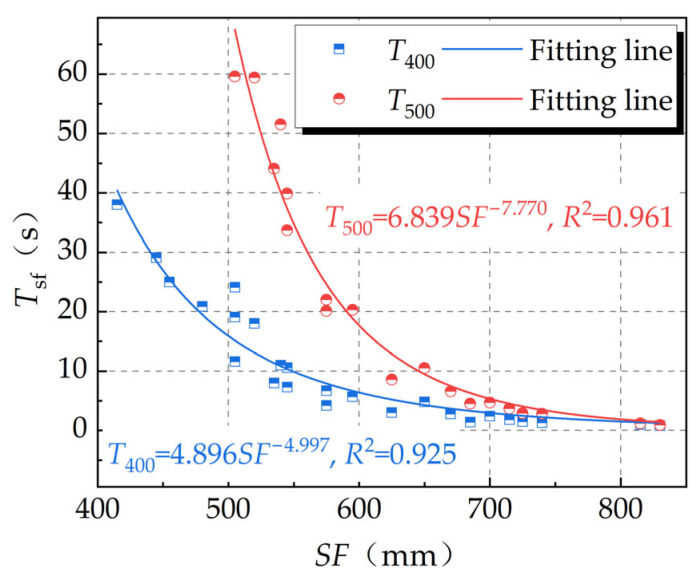
Relationships of flow time with slump flow of fresh UNC.

Regarding the workability of the fresh concrete, the slump and slump-flow ranged within 240–280 mm and 415–830 mm, respectively. The flow times *T*_400_ and *T*_500_ varied within 1.0–38 s and 0.9–59.6 s, respectively. As shown in Figure 2, a negative correlation was presented between the flowability and the flow time, and the difference between *T*_400_ and *T*_500_ became larger when the flowability was smaller. This was because of the smaller flowability of the fresh concrete, along with a higher plastic viscosity and yield stress [36,37]. A decrease in the flowability of the fresh concrete resulted in a significant prolonging of the flow time, which reached a larger slump-flow [38,39].

It can be seen that No. 23 UNC had the best workability, with the slump, slump flow, *T*_400_, and *T*_500_ reaching 280 mm, 830 mm, 0.8 s, and 0.9 s, respectively, denoted by *A*_5_*B*_3_*C*_2_*D*_1_*E*_5_*F*_4_. This corresponded to a water/binder ratio of 0.52 (level 5), sand ratio of 50% (level 3), maximum aggregate size of 16 mm (level 2), dispersant dosage of 1.0% (level 1), water reducer dosage of 1.6% (level 5), and fly ash content of 30% (level 4). Therefore, the target workability of the fresh UNC could be realized using appropriate mix proportion.

**Figure 3:**
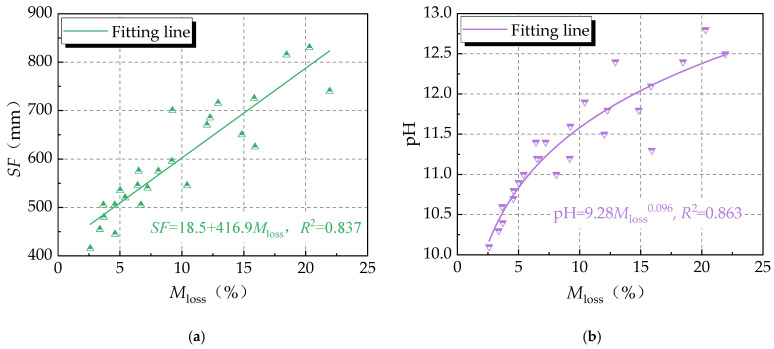
Relationships of performance indicators of UNC: (**a**) slump flow and washout loss; and (**b**) pH value and washout loss.

Regarding the dispersion resistance, the washout loss rate and pH value ranged within 2.6–21.9% and 10.1–12.8, respectively. As shown in Figure 3, the washout loss rate increased linearly with the slump-flow, with a correlation coefficient of 0.83. This was due to the better flowability of fresh concrete being associated with a relatively higher paste content, and a tendency for higher segregation [36,37]. Meanwhile, a positive exponential correlation existed between the washout loss rate and the pH value, with a correlation coefficient of 0.86. This meant that a greater content of binder paste came into the water to rise the alkalinity of the distilled water that was used for the testing, resulting in an increased pH value. From the test results in Table 4, No. 5 UNC had the best dispersion resistance, denoted as *A*_1_*B*_5_*C*_5_*D*_5_*E*_5_*F*_5_, with a washout loss rate of 2.6% and a pH value of 10.1.

**Figure 4:**
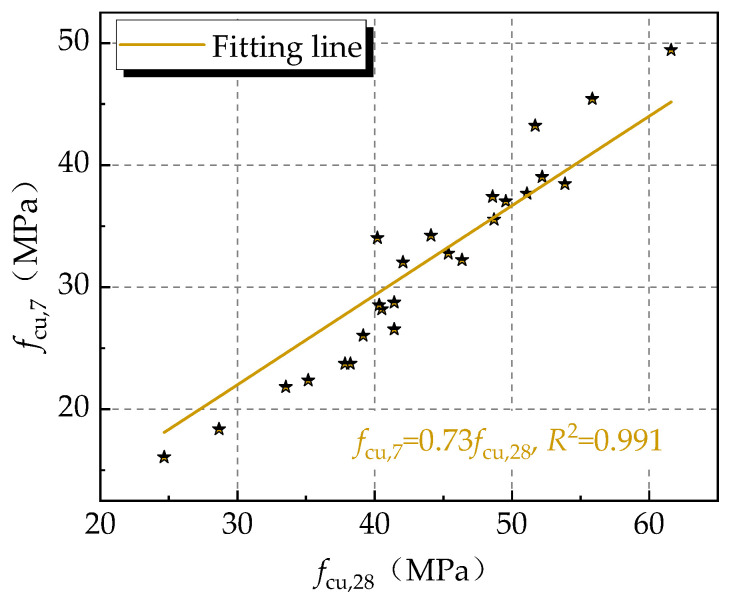
Relationships of the compressive strength of UNC at 7 d and 28 d.

For the compressive strength of the UNC, a similar variation was presented at the curing ages of 7 days and 28 days. As shown in Figure 4, a formula can be obtained using a linear fitting for the compressive strengths at 7 days and 28 days, with a ratio of 0.73 and a correlation coefficient of 0.91. This was similar to the strength development of the conventional concrete at the curing age from 7 days to 28 days [22,40]. No. 1 UNC provided the highest compressive strengths at 7 days and 28 days, denoted as *A*_1_*B*_1_*C*_1_*D*_1_*E*_1_*F*_1_, with strengths reaching 49.4 MPa and 61.6 MPa, respectively.

### 3.2. Range and Variance Analysis

Range and variance methods were used to analyze the workability, anti-dispersion, and compressive strength of the UNC [41,42]. In the range analysis, the range (*R*) is represented by *R* = max(*k*_i_) − min(*k*_i_), where *k*_i_ represents the average value of a certain indicator at the *i*th level of each factor and *i* represents the level of the factor from 1 to 5. A larger range value for a factor in relation to a certain target indicates a greater influence of that factor on the target, i.e., a higher sensitivity. Variance can better analyze the significance level of each factor on the target, which includes the sum of squares of deviations (*SS*), degree of freedom (*d*_f_), mean squares of deviations (*MS*), *F*-value, and significance level. In a variance analysis, it is necessary to calculate the error, which can be calculated by Equations (1)–(3). In this study, the *SS,* with a minimum value for each factor under each target analysis, was considered as the error *SS*. The statistical definition of the threshold for the *F*-value was *F*_0.01_(4, 4) = 15.98 and *F*_0.05_(4, 4) = 6.39. When the *F*-value was greater than 15.98, the factor was considered to have a very significant level on the target, marked by “★★”; when the *F*-value was greater than 6.39 but less than 15.98, the factor was considered to have a significant level on the target, represented by ”★”.

(1) $$SS=\frac{n}{r}\sum_{i=1}^{r}\left(k_{i}-\bar{k}\right)$$

(2) $$MS=\frac{SS}{d_{f}}$$

(3) $$F=\frac{MS}{MS_{e}}$$

#### 3.2.1. Workability

Table 5 shows the results of the range and variance calculations for the various workability indicators. It can be seen that the *S*, *SF*, and *T*_400_ sensitivity are ranked in the order of *DACBEF*, *ADCFBE*, and *ADECFB* from high to low. The factor with the largest range in slump was the dosage of dispersant *D*, followed by the water–binder ratio factor *A*. The factor with the largest range in the spread was the *w/b* factor *A*, followed by the dosage of dispersant *D*. The factor with the largest range in flow time *T*_400_ was the water/binder ratio factor *A*, followed by the dosage of dispersant *D*. Factors *B*, *C*, *E*, and *F* had relatively small sensitivities to the three performance indicators. A higher slump and slump-flow induced a lower flow time. Therefore, the optimal combinations of the slump, slump flow, and flow time were *A*_5_*B*_3_*C*_1_*D*_1_*E*_3_*F*_3_, *A*_5_*B*_3_*C*_5_*D*_1_*E*_4_*F*_5_, and *A*_5_*B*_4_*C*_1_*D*_1_*E*_3_*F*_1_.

Based on the test results of the significance level in the variance analysis, the order of factors that significantly affected the slump, slump-flow, and *T*_400_ were *DACBEF*, *DACBFE*, and *ADCEFB*, respectively. The factors *A* and *D* presented a significant effect on the slump, slump-flow, and *T*_400_, while the other four factors had a slight impact on these performances.

**Figure 5:**
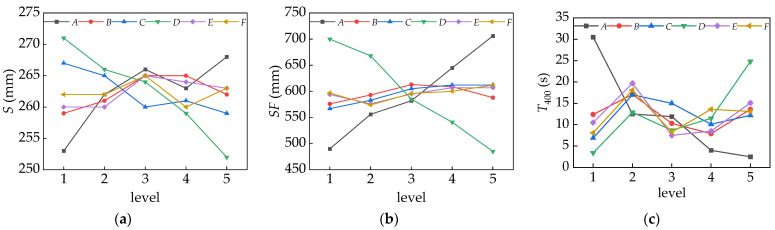
Various trends in the mean values *k*_i_ of properties at different factor levels: (**a**) slump; (**b**) slump flow; and (**c**) *T*_400_.

Figure 5 shows the trends of the average values of the various performance indicators at different levels of factors. The increase in the water/binder ratio contributed to increasing the slump and slump-flow and decreasing the flow time, which was beneficial to the flowability of the fresh concrete. In this study, the slump increased from 253 mm to 268 mm, the slump-flow increased from 490 mm to 706 mm, and the flow time decreased from 30.5 s to 2.5 s. The increase in the sand ratio benefitted the slump and slump-flow, due to the amount of mortar coating the aggregates being increased to provide good lubrication. The slump was reduced with an increase in the particle size of the coarse aggregate due to the viscosity of the fresh UNC being increased with a strong skeleton of crushed limestones, while the slump-flow was increased to some extent due to the yield stress of the fresh UNC being reduced with an increased surface area to volume ratio. The increase in the dispersion resistance agent reduced the slump and slump-flow and prolonged the flow time. In this condition, the slump decreased from 271 mm to 252 mm, the slump-flow decreased from 700 mm to 485 mm, and the flow time increased from 3.4 s to 24.8 s. It was apparent that the addition of the dispersion resistance agent increased the viscosity and yield stress of the fresh concrete. The effects of the water reducer and fly ash on the flowability can be neglected.

#### 3.2.2. Non-Dispersible Performance

Table 6 shows the calculation results of the range and variance of the non-dispersible performance indicators. The sensitivity of each factor to the *M*_loss_ and pH value, from high to low, was *DACFBE* and *DAFBCE*. Therefore, *D* was the factor with a largest range of *M*_loss_ and pH values, followed by the factor A. The sensitivity of factors *BCEF* to the *M*_loss_ and pH value was lower. In terms of non-dispersible performance, both the *M*_loss_ and pH values should be as small as possible, and the optimal combinations of *M*_loss_ and pH value were *A*_1_*B*_3_*C*_1_*D*_5_*E*_2_*F*_1_ and *A*_1_*B*_5_*C*_5_*D*_5_*E*_2_*F*_3_.

Based on the variance analysis, the significance levels of the factors affecting the *M*_loss_ and pH value were in the order of *DACFBE* and *DAFBCE*. This was the same as the order of sensitivity. Therefore, both *A* (water/binder ratio) and *D* (dosage of dispersion resistance agent) had a very significant influence on the *M*_loss_ and pH value, while the other four factors had no slight impact.

**Figure 6:**
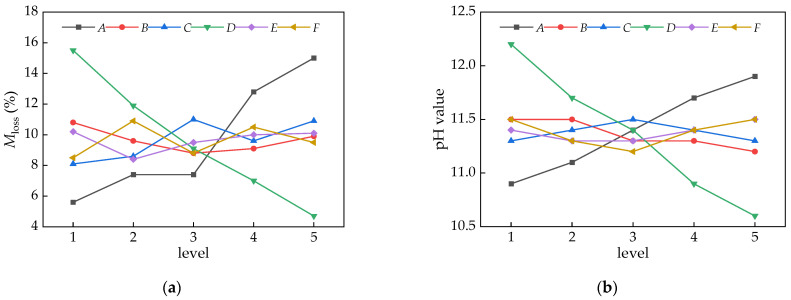
Various trends in the mean values *k*_i_ of anti-dispersion at different factor levels: (**a**) *M*_loss_; and (**b**) pH value.

Figure 6 shows the trend of the average values of the various anti-dispersion performance indicators at different factor levels. It can be seen that, with an increase in the water/binder ratio, both the *M*_loss_ loss and pH value increased significantly. This was because the increase in the water/binder ratio led to a volume increase in the binder paste, which was easily washed out of the fresh UNC.

Relatively, the factors of sand ratio, coarse aggregate particle size, water reducer, and fly ash had a smaller impact on the dispersion resistance, with *M*_loss_ varying by 2.0%, 2.9%, 1.7%, and 2.4%, respectively, and the pH value varying by 0.26, 0.23, 0.19, and 0.32, respectively. Obviously, with an increase in the dosage of the dispersion resistance agent, both the *M*_loss_ and pH value showed a linear decrease. *M*_loss_ decreased from 15.5% to 4.7%, and the pH value decreased from 12.2 to 10.6. This was because of the dispersion resistance agent being composited mainly with high molecular polysaccharides, which have an adsorption capacity and bridging effect, thereby increasing the viscosity of the binder paste [43,44].

#### 3.2.3. Compressive Strength

The results of the range and variance analyses for the compressive strength are summarized in Table 7. The sensitivity of the various factors from high to low on the compressive strengths at the curing ages of 7 days and 28 days was *AFECDB* and *AFCBDE*, respectively. The factor with the largest range for the compressive strength was factor A (the water/binder ratio), followed by factor *F* (the amount of fly ash). Factors *BCDE* presented a lower sensitivity to the compressive strength. Due to a higher strength being preferred in terms of mechanical properties, the optimal combinations for the compressive strengths at the curing ages of 7 days and 28 days were *A*_1_*B*_4_*C*_1_*D*_1_*E*_2_*F*_1_ and *A*_1_*B*_1_*C*_1_*D*_4_*E*_3_*F*_1_, respectively.

Through the analysis of variance and significance level testing, the significance levels for the compressive strengths at the curing ages of 7 days and 28 days were *AFECDB* and *AFCBED*, respectively. Therefore, both factor *A* and factor *F* were very significant, having an influence on the compressive strength, while the other four factors were insignificant.

**Figure 7:**
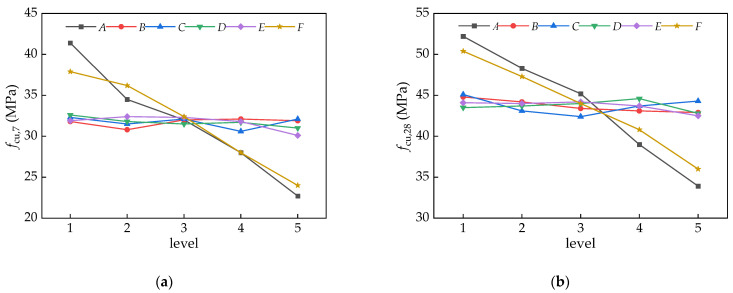
Various trends in the mean values *k*_i_ of compressive strength at different factor levels: (**a**) *f*_cu,7_; and (**b**) *f*_cu,28_.

Figure 7 shows the trend of the average values of the compressive strength at different factor levels. With the increase in the water/binder ratio (factor *A*), the compressive strengths at the curing ages of 7 days and 28 days tended to decrease. With the water/binder ratio increasing from 0.38 to 0.52, the compressive strength at the curing age of 7 days decreased from 41.4 MPa to 22.7 MPa, with a decrement of 18.7 MPa, while that at the curing age of 28 days decreased from 52.2 MPa to 33.9 MPa with a decrement of 18.3 MPa. Meanwhile, with the fly ash content increasing from 0 to 40%, the compressive strength at the curing age of 7 days decreased from 37.9 MPa to 24.0 MPa, while that at the curing age of 28 days decreased from 40.4 MPa to 36.0 MPa. The effects of the other four factors on the compressive strength were relatively small.

### 3.3. Comprehensive Optimization Analysis

According to the requirements of self-compacting UNC, its workability should be firstly satisfied with a reasonable slump flowability to be easily constructed, while the its anti-dispersion performance should be higher to reduce the wash-out rate of fresh UNC and ensure a high quality of UNC underwater pouring. Meanwhile, the mechanical performance should reach the target of hardened UNC. Generally, the key indices are those that the slump-flow needs to be above 550 mm [33], the erosion loss rate should be below 10%, and the water-to-land compressive strength ratio should be above 0.7 [35]. Based on the test results of this study, the UNC of numbers 10, 13, 18, and 22 met the above requirements.

**Figure 8:**
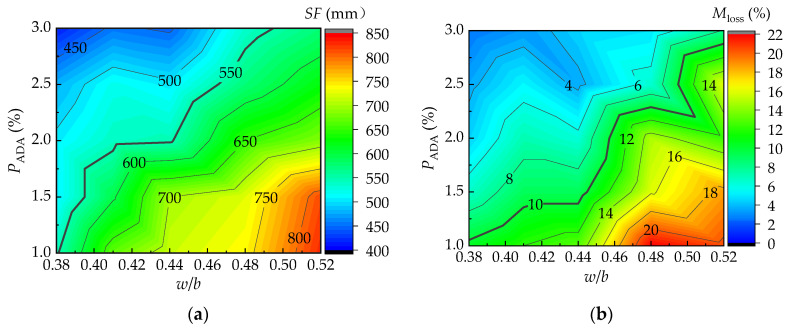
Comprehensive impact of test factors on workability and anti-dispersion performance: (**a**) effect of *w/b* and *P*_ADA_ on *SF*; and (**b**) effect of *w/b* and *P*_ADA_ on *M*_loss_.

Combined with the results of the range and variance analysis, the water/binder ratio and the content of the dispersion resistance agent were the main factors affecting the workability and anti-dispersion performance of the UNC. This is similar to previous studies [11,45,46,47]. As shown in Figure 8, the slump-flow and the washout loss rate had certain relationships with the water/binder ratio and the content of the dispersion resistance agent.

**Figure 9:**
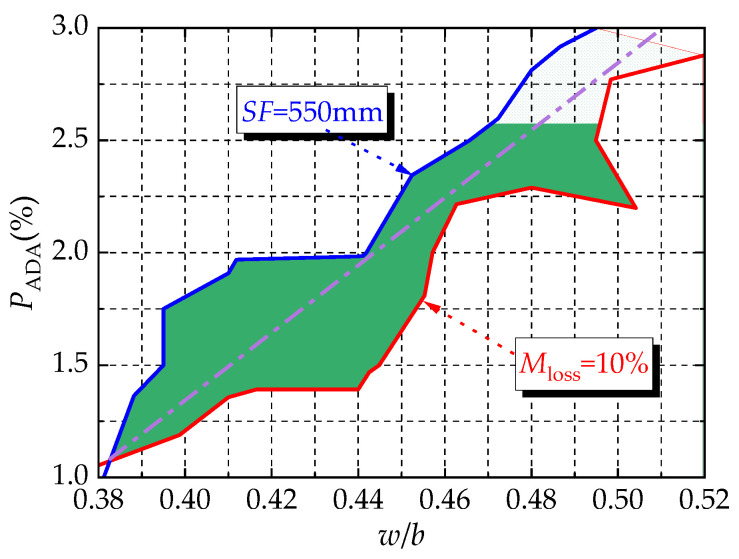
Coupling effect of *w/b* and *P*_ADA_ on *SF* and *M*_loss_.

Within the range where the slump-flow was greater than 550 mm and the washout loss rate was less than 10%, there were certain ranges of variation in the water/binder ratio and the content of the dispersion resistance agent. This made the fresh UNC meet the requirements, as shown by the shadow part in Figure 9. After a joint fitting of the test results, Formulas (4) and (5) are proposed to predict the slump-flow and washout loss rate considering the influences of the water/binder ratio and the content of the dispersion resistance agent, with correlation coefficients of 0.949 and 0.892.

(4) $$SF=3162.6w/b+11203.9P_{\mathrm{ADA}}-798.3{\left(w/b\right)}^{2}-25714.3{\left(P_{\mathrm{ADA}}\right)}^{2}-47792.2(w/b\times P_{\mathrm{ADA}})$$

(5) $$M_{\mathrm{loss}}=-95.5w/b+55.1P_{\mathrm{ADA}}+232.6{\left(w/b\right)}^{2}+9828.6{\left(P_{\mathrm{ADA}}\right)}^{2}-2187.0(w/b\times P_{\mathrm{ADA}})+19.4$$

**Figure 10:**
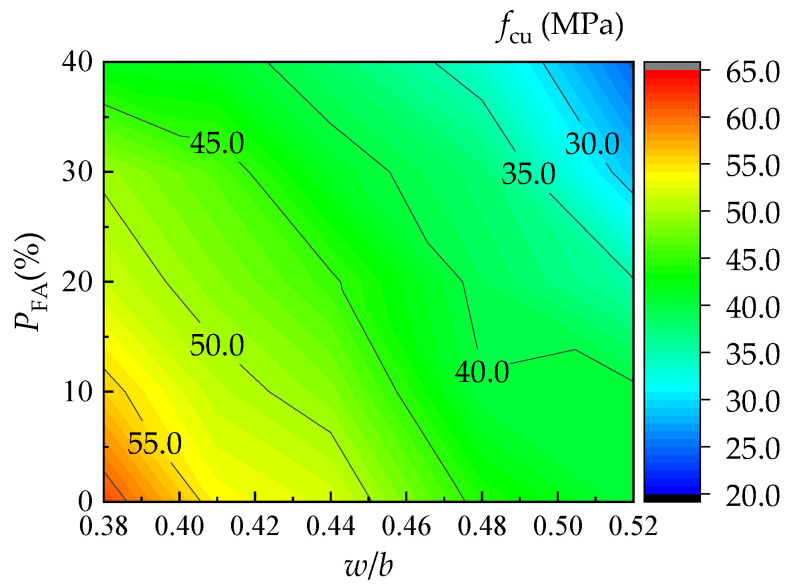
Comprehensive impact of *w/b* and *P*_FA_ on *f*_cu,28_.

Figure 10 shows the effect of the water/binder ratio and fly ash content on the compressive strength of the UNC at the curing age of 28 days. A formula is proposed for predicting the compressive strength based on the joint fitness of the test results of this study, with a correlation coefficient of 0.968.

(6) $$f_{\mathrm{cu}}{}_{,28}=-58.5w/b-36.3P_{\mathrm{FA}}-86.2{\left(w/b\right)}^{2}+-23.6(P_{\mathrm{FA}}{)}^{2}23.2{\left(w/b\times P_{\mathrm{FA}}\right)}^{2}+93.8$$

## 4. Conclusions

(1) The slump flowability of the fresh UNC had strong correlation with the flow time. With a decrease in the slump-flow, the flow time significantly grew with an exponential, however, the washout loss rate presented a linear reduction and an increase in the pH value. The optimal workability of the fresh UNC could be given with a slump, slump flow, and *T*_500_ of 280 mm, 830 mm, and 0.9 s. The washout loss rate of the fresh UNC could be limited to around 10% using a reasonable content of dispersion resistance agent.
(2) According to the range and variance analysis, among the factors, including the water/binder ratio, the sand ratio, the particle size of the coarse aggregate, the content of the dispersion resistance agent, the content of the water reducer agent, and the dosage of fly ash, the water/binder ratio and the content of dispersion resistance agent were the main factors affecting the workability and dispersibility of the UNC, while the water/binder ratio and dosage of fly ash were the main factors influencing the compressive strength of the UNC.
(3) The compressive strength of the UNC at the curing age of 7 days was approximately 73% of that at the curing age of 28 days.
(4) With the joint fitness of the test results, formulas were proposed to predict the slump-flow and the washout loss rate of fresh UNC with the factors of the water/binder ratio and the content of the dispersion resistance agent. Meanwhile, a formula for predicting the compressive strength at the curing age of 28 days was also proposed considering the influence of the water/binder ratio and the dosage of fly ash.

## Figures and Tables

**Table 1:** Chemical compositions of cement and fly ash.

Compositions	Percent of Chemical Composition (%)						
	CaO	SiO_2_	MgO	Al_2_O_3_	Fe_2_O_3_	SO_3_	LOI
Cement	62.3	19.7	2.3	5.3	3.4	3.3	3.7
Fly ash	6.2	59.2	3.8	20.1	5.3	3.1	2.3

**Table 2:** The factors and levels for orthogonal test.

Level	Factors					
	A	B	C	D	E	F
	w/b	*βs* (%)	*dca* (mm)	*PDRA* (%)	*PSP* (%)	*PFA* (%)
①	0.38	40	10	1.0	0.8	0
②	0.41	45	16	1.5	1.0	10
③	0.44	50	20	2.0	1.2	20
④	0.48	55	25	2.5	1.4	30
⑤	0.52	60	31.5	3.0	1.6	40

**Table 3:** Level of each parameter for the orthogonal test design—L25 (5^6^).

Factor	Trial Number																								
	1	2	3	4	5	6	7	8	9	10	11	12	13	14	15	16	17	18	19	20	21	22	23	24	25
*A*	①	①	①	①	①	②	②	②	②	②	③	③	③	③	③	④	④	④	④	④	⑤	⑤	⑤	⑤	⑤
*B*	①	②	③	④	⑤	①	②	③	④	⑤	①	②	③	④	⑤	①	②	③	④	⑤	①	②	③	④	⑤
*C*	①	②	③	④	⑤	②	③	④	⑤	①	③	④	⑤	①	②	④	⑤	①	②	③	⑤	①	②	③	④
*D*	①	②	③	④	⑤	③	④	⑤	①	②	⑤	①	②	③	④	②	③	④	⑤	①	④	⑤	①	②	③
*E*	①	②	③	④	⑤	④	⑤	①	②	③	②	③	④	⑤	①	⑤	①	②	③	④	③	④	⑤	①	②
*F*	①	②	③	④	⑤	⑤	①	②	③	④	④	⑤	①	②	③	③	④	⑤	①	②	②	③	④	⑤	①

**Table 4:** Test results of the orthogonal experiment for UNC.

No.	Workability				Anti-Dispersion		Compressive Strength	
	*S* (mm)	*SF* (mm)	*T*_sf_ (s)		Washout Loss (%)	pH	*f*_cu_ (MPa)	
			*T*_400_	*T*_500_			7 d	28 d
1	260	545	10.6	39.9	10.4	11.9	49.4	61.6
2	255	505	24.1	-	6.7	11.2	45.4	55.9
3	260	505	19.1	-	4.6	10.7	43.2	51.7
4	250	480	20.9	-	3.7	10.4	37.0	49.6
5	240	415	38.0	-	2.6	10.1	32.0	42.1
6	265	540	11.0	51.5	7.2	11.4	26.5	41.4
7	255	520	18.0	59.4	5.4	11.0	38.5	53.9
8	250	455	25.0	-	3.4	10.3	37.7	51.1
9	270	670	2.8	6.6	12.0	11.5	37.4	48.6
10	270	595	5.7	20.3	9.2	11.2	32.2	46.4
11	245	445	29.1	-	4.6	10.8	28.7	41.4
12	275	715	1.8	3.7	12.9	12.4	23.7	38.2
13	270	700	2.4	4.7	9.2	11.6	39.0	52.2
14	275	545	7.3	33.7	6.4	11.4	35.5	48.7
15	265	505	11.6	59.6	3.7	10.6	32.8	45.4
16	265	725	1.5	2.9	15.8	12.1	26.0	39.2
17	255	650	4.8	10.5	14.8	11.8	23.7	37.8
18	265	575	4.2	20.1	6.5	11.2	21.8	33.5
19	260	535	8.0	44.1	5.0	10.9	34.2	44.1
20	270	740	1.3	2.8	21.9	12.5	34.0	40.2
21	260	625	3.0	8.6	15.9	11.3	28.2	40.5
22	265	575	6.7	22.0	8.1	11.0	22.4	35.2
23	280	830	0.8	0.9	20.3	12.8	18.4	28.7
24	270	815	1.0	1.2	18.5	12.4	16.1	24.7
25	265	685	1.4	4.5	12.3	11.8	28.5	40.3

**Table 5:** The range and variance analyses of the slump, slump flow, and *T*_400_ of fresh UNC.

Factors	*S* (mm)						*SF* (mm)						*T*_400_ (s)					
	*A*	*B*	*C*	*D*	*E*	*F*	*A*	*B*	*C*	*D*	*E*	*F*	*A*	*B*	*C*	*D*	*E*	*F*
*k*_1_	253	259	267	271	260	262	490	576	567	700	594	597	30.5	12.4	6.9	3.4	10.5	8.1
*k*_2_	262	261	265	266	260	262	556	593	583	668	576	574	12.5	17.1	17.0	12.9	19.7	18.1
*k*_3_	266	265	260	264	265	265	582	613	605	585	595	596	11.9	10.3	15.0	8.7	7.5	8.3
*k*_4_	263	265	261	259	264	260	645	609	612	541	607	600	4.0	7.9	10.1	11.5	8.5	13.6
*k*_5_	268	262	259	252	263	263	706	588	612	485	607	612	2.5	13.6	12.2	24.8	15.1	13.1
*R*	15	6	8	19	5	5	216	37	45	215	31	38	28.1	9.2	10.1	21.4	12.2	10.1
Ranking	*D > A > C > B > E = F*						*A > D > C > F > B > E*						*A > D > E > C > F > B*					
*SS*	666	136	236	1046	106	66	137,664	4654	8014	157,334	3234	3784	1269.9	40.5	117.4	922.9	116.4	83.7
*d*_f_	4	4	4	4	4	4	4	4	4	4	4	4	4	4	4	4	4	4
*F*-value	10.1	2.1	3.6	15.8	1.6	1.0	42.6	1.4	2.5	48.6	1.0	1.2	31.4	1.0	2.9	22.8	2.99	2.1
Significance	★			★			★★			★★			★★			★★		

Note: “★” represents that the factor had an effect at significant level on the target, “★★”; represents that the factor had a an effect at very significant level on the target.

**Table 6:** The range and variance analyses of the *M*_loss_ and pH value of fresh UNC.

Factors	*M*_loss_ (%)						pH Value					
	*A*	*B*	*C*	*D*	*E*	*F*	*A*	*B*	*C*	*D*	*E*	*F*
*k*_1_	5.6	10.8	8.1	15.5	10.2	8.5	10.9	11.5	11.3	12.2	11.4	11.5
*k*_2_	7.4	9.6	8.6	11.9	8.4	10.9	11.1	11.5	11.4	11.7	11.3	11.3
*k*_3_	7.4	8.8	11.0	9.1	9.5	8.8	11.4	11.3	11.5	11.4	11.3	11.2
*k*_4_	12.8	9.1	9.6	7.0	10.0	10.5	11.7	11.3	11.4	10.9	11.4	11.4
*k*_5_	15.0	9.9	10.9	4.7	10.1	9.5	11.9	11.2	11.3	10.6	11.5	11.5
*R*	9.4	2.0	2.9	10.8	1.7	2.4	1.00	0.26	0.23	1.60	0.19	0.32
Ranking	*D > A > C > F > B > E*						*D > A > F > B > C > E*					
*SS*	326.1	12.0	34.4	353.4	10.8	21.5	3.47	0.26	0.14	8.08	0.12	0.31
*d*_f_	4	4	4	4	4	4	4	4	4	4	4	4
*F*-value	30.3	1.1	3.2	32.8	1.0	2.0	28.2	2.1	1.1	65.7	1.0	2.5
Significance	★★			★★			★★			★★		

Note: “★★” represents that the factor had a an effect at very significant level on the target.

**Table 7:** The range and variance analyses of the compressive strength of UNC.

Factors	7 d Compressive Strength (MPa)						28 d Compressive Strength (MPa)					
	*A*	*B*	*C*	*D*	*E*	*F*	*A*	*B*	*C*	*D*	*E*	*F*
*k*_1_	41.4	31.8	32.3	32.6	31.9	37.9	52.2	44.8	45.1	43.5	44.1	50.4
*k*_2_	34.5	30.8	31.5	31.8	32.4	36.2	48.3	44.2	43.1	43.7	44.0	47.3
*k*_3_	32.0	32.0	32.1	31.5	32.3	32.4	45.2	43.4	42.4	44.0	44.2	44.0
*k*_4_	28.0	32.1	30.6	31.7	31.8	28.0	39.0	43.1	43.7	44.6	43.7	40.8
*k*_5_	22.7	31.9	32.1	31.0	30.1	24.0	33.9	42.9	44.3	42.8	42.5	36.0
*R*	18.7	1.3	1.7	1.6	2.3	13.9	18.3	2.0	2.7	1.8	1.7	14.4
Ranking	*A > F > E > C > D > B*						*A > F > C > B > D > E*					
*SS*	985.5	6.0	9.6	6.5	17.6	657.9	1068.4	13.0	21.6	8.9	9.7	630.5
*d*_f_	4	4	4	4	4	4	4	4	4	4	4	4
*F*-value	165.6	1.0	1.6	1.1	3.0	110.6	120.6	1.5	2.4	1.0	1.1	71.2
Significance	★★					★★	★★					★★

Note: “★★” represents that the factor had a an effect at very significant level on the target.

## Data Availability

Data are available with the first author and can be shared with anyone upon reasonable request.

## References

[1] Xia Z., Lin L., Zhang J., Jiang S. (2022). Seismic performance of underwater RC bridge piers strengthened with self-compacting concrete-filled BFRP jacket. Structures.

[2] Lu H., Sun X., Ma H.Y. (2022). Anti-washout Concrete: An overview. Constr. Build. Mater..

[3] Geng H., Ding X., Du H., Shi J., Li C., Li X. (2022). Application of self-compacting steel fiber reinforced concrete for pervious frames used for river revetment. Appl. Sci..

[4] Jiao L. (2022). Study on corrosion resistance of grouting materials for subsea tunnels and its engineering application. Modern Tunnel. Technol..

[5] Xu J., Wang H. (2022). Numerical analysis of seepage characteristics and stability of cofferdam in east artificial island of Shenzhen-Zhongshan link during pumping. Tunnel Constr..

[6] Zhang K., Duan Y., Liu X., Shi Z. (2021). Study on underwater quick repairing of lining slab. Yangtze River.

[7] Jin H., Wei Y., Zhang Y., Huang Z., Liu L. (2023). Compressive performance of underwater concrete columns strengthened by nondispersive mortar and stainless steel tubes. Case Studies Constr. Mater..

[8] Yu S.Q., Bao S. (2013). Innovation and application of one-step forming technology under water of varied-section drilling pile. Port Waterway Eng..

[9] Hu H., Zhou Y., Zhang J. (2021). Key base sealing techniques for north main pier steel cofferdam of Qingshan Changjiang River Highway bridge in Wuhan. Bridge Constr..

[10] Jeon I.K., Woo B.H., Yoo D.H., Ryou J.S., Kim H.G. (2021). Evaluation of the hydration characteristics and anti-washout resistance of non-dispersible underwater concrete with nano-SiO_2_ and MgO. Materials.

[11] Wang Y., Chen S., Qiu L., Nasr A.A., Liu Y. (2023). Experimental study on the slump-flow underwater for anti-washout concrete. Constr. Build. Mater..

[12] Zhang Y., Dong Y. (2021). Effect of fly ash and silica fume on the performance of underwater self-compacting concrete. Concrete.

[13] Dong Y., Zhang Y. (2020). Study on mixing ratio design and performance of non-dispersible underwater concrete for channel lining slab. Water Resou. Hydropower Eng..

[14] Heniegal A.M.A., Maaty A.A.E.S., Agwa I.S. (2016). Influence of anti washout admixtures and coarse aggregate types on self-flowing underwater concrete properties. Intern. J. Civ. Struct. Eng..

[15] Assaad J.J., Issa C.A. (2013). Mechanisms of strength loss in underwater concrete. Mater. Struct..

[16] Wen Y.X., Zhou W., Tang J.W. (2021). Study on anti-scouring performance of freshly poured anti-washout concrete. Water Resour. Hydropower Eng..

[17] Zaidi F.H.A., Ahmad R., Abdullah M.M.A.B., Rahim S.Z.A., Yahya Z., Li L., Ediati R. (2021). Geopolymer as underwater concreting material: A review. Constr. Build. Mater..

[18] Zhang Y., Sun G., Zhang G., Wang C., Wang Y. (2022). Optimization and prediction for anti-washout ability of underwater concrete based on factorial design. J. Build. Mater..

[19] Zhang M., Zhou M., Huang J., Yang D. (2016). Flocculants and adjuvants on shear stress and viscosity of cement paste. Bulletin Chinese Ceramic Soci..

[20] Nasr A.A., Chen S., Wang Y., Jin F., Qiu L. (2022). Strength evaluation of a new underwater concrete type. Case Studies Constr. Mater..

[21] Kumar B.G., Muthu M., Chajec A., Sadowski L., Govindaraj V. (2022). The effect of silica fume on the washout resistance of environmentally friendly underwater concrete with a high-volume of siliceous fly ash. Constr. Build. Mater..

[22] Li C., Wang F., Deng X., Li Y., Zhao S. (2019). Testing and prediction of the strength development of recycled-aggregate concrete with large particle natural aggregate. Materials.

[23] Ma J., Wang D., Zhao S., Duan P., Yang S. (2021). Influence of particle morphology of ground fly ash on the fluidity and strength of cement paste. Materials.

[24] Li C., Geng H., Zhou S., Dai M., Sun B., Li F. (2022). Experimental study on preparation and performance of concrete with large content of fly-ash. Front. Mater..

[25] Liu S., Zhu M., Ding X., Ren Z., Zhao S., Zhao M., Dang J. (2021). High-durability concrete with supplementary cementitious admixtures used in corrosive environment. Crystals.

[26] Qu F., Zhang J., Liu G., Zhao S. (2022). Experimental study on chloride-ion diffusion in concrete affected by exposure conditions. Materials.

[27] (2007). Common Portland Cement.

[28] (2014). Technique Code for Application of Fly Ash Concrete.

[29] (2006). Standard for Technical Requirements and Test Method of Sand and Crushed Stone (or Gravel) for Ordinary Concrete.

[30] Zhao M., Ding X., Li J., Law D. (2018). Numerical analysis of mix proportion of self-compacting concrete compared to ordinary concrete. Key Eng. Mater..

[31] Ding X., Zhao M., Qiu X., Wang Y., Ru Y. (2022). The optimization of mix proportion design for SCC: Experimental study and grey relational analysis. Materials.

[32] (2016). Standard for Test Method of Performance on Ordinary Fresh Concrete.

[33] (2012). Technical Specification for Application of Self-compacting Concrete.

[34] (1989). Test Method for Determining the Resistance of Freshly-Mixed Concrete to Washing-Out in Water.

[35] (2021). Test Code on Anti-Washout Underwater Concrete.

[36] Zhao M., Dai M., Li J., Li C. (2023). Case study on performance of pumping concrete with super-fine river-sand and manufactured-sand. Case Stud. Constr. Mater..

[37] Zhao M., Li C., Li J., Yue L. (2023). Experimental study on performance of steel fiber reinforced concrete for remote-pumping construction. Materials.

[38] Khayat K.H., Yahia A., Sonebi M. (1999). Applications of statistical models for proportioning underwater concrete. ACI Mater. J..

[39] Yahia A., Khayat K.H. (2001). Experiment design to evaluate interaction of high-range water-reducer and antiwashout admixture in high-performance cement grout. Cem. Concr. Res..

[40] Ding X., Li C., Xu Y., Li F., Zhao S. (2016). Experimental study on long-term compressive strength of concrete with manufactured sand. Constr. Build. Mater..

[41] Ge Z., Gao Z., Sun R., Zheng L. (2012). Mix design of concrete with recycled clay-brick-powder using the orthogonal design method. Constr. Build. Mater..

[42] Li J., Yang F., Zhang H., Wu Z., Tian Y., Hou X., Xu Y., Ren J. (2020). Comparative analysis of different valve timing control methods for single-piston free piston expander-linear generator via an orthogonal experimental design. Constr. Build. Mater..

[43] Xiao S., Zhang M., Zou D., Liu T., Zhou A., Li J. (2023). Influence of seawater and sea sand on the performance of Anti-washout underwater concrete: The overlooked significance of Mg^2+^. Constr. Build. Mater..

[44] Sikandar M.A., Wazir N.R., Khan A., Nasir H., Ahmad W., Alam M. (2020). Effect of various anti-washout admixtures on the properties of non-dispersible underwater concrete. Constr. Build. Mater..

[45] Sun G., Wang P., Zhang Y., Yan N. (2021). Research progress on performance of anti-washout underwater concrete. Mater. Rep..

[46] Sun G., Zhang Y., Yan N., Wang Y., Li Z. (2022). Study progress on design and characterization of anti-washout ability of anti-washout underwater concrete. Mater. Rep..

[47] Govin A., Bartholin M., Schmidt W., Grosseau P. (2019). Combination of superplasticizers with hydroxypropyl guar, effect on cement-paste properties. Constr. Build. Mater..

